# Effectiveness of Traditional vs. Modern Contraception According to the 2017 IDHS: The Urgent Need for Rights-Based Counseling in Indonesia’s Family Planning Program

**DOI:** 10.3390/ijerph23050643

**Published:** 2026-05-12

**Authors:** Siti Dariyani, Budi Utomo, Eflita Meiyetriani, Sudibyo Alimoeso, Maria Gayatri, Sukma Rahayu, Restu Octasila

**Affiliations:** 1 Doctoral Program in Public Health, Faculty of Public Health, Universitas Indonesia, Depok 16424, Indonesia; restuoctasila@gmail.com; 2 Department of Population and Biostatistics, Center for Health Research, Faculty of Public Health, Universitas Indonesia, Depok 16424, Indonesia; budi.utomo.ui@gmail.com (B.U.); sukmarahayu47@gmail.com (S.R.); 3 Southeast Asian Ministers of Education Organization Regional Centre for Food and Nutrition (SEAMEO RECFON), East Jakarta 13120, Indonesia; eflita@seameo-recfon.org; 4 Indonesian Demographic Association, Jakarta 13610, Indonesia; salimoeso5511@gmail.com; 5 Ministry of Population and Family Planning Development/National Population and Family Planning Board, Jakarta 13610, Indonesia; maria.gayatri.bkkbn@gmail.com

**Keywords:** sociodemographic, pregnancy risk, dropout, traditional contraception

## Abstract

**Highlights:**

**Public health relevance—How does this work relate to a public health issue?**

- The increasing use of traditional contraceptive methods in Indonesia raises concerns about the risk of unintended pregnancy amid stagnating modern contraceptive use.
- Understanding contraceptive effectiveness and discontinuation patterns is essential to inform family planning strategies and improve reproductive health outcomes.

**Public health significance—Why is this work of significance to public health?**

- Women using traditional contraception had a significantly higher risk of pregnancy compared with modern contraceptive users, despite similar discontinuation rates.
- Traditional contraceptive use was more prevalent among older, more educated, higher socioeconomic status women, and those living in urban areas.

**Public health implications—What are the key implications or messages for practitioners, policy makers and/or researchers in public health?**

- Family planning programs should strengthen comprehensive counseling to support informed contraceptive choices while respecting reproductive autonomy.
- Improving service quality and addressing contraceptive discontinuation are essential to enhance the effectiveness of modern methods and reduce unintended pregnancies.

**Abstract:**

Introduction: The increasing use of traditional contraceptive methods in Indonesia may raise the risk of unintended pregnancies, while modern contraceptive use has stagnated and often experiences discontinuation. Nevertheless, contraceptive choice is an individual right and should guide family planning programs. This study aimed to estimate contraceptive effectiveness based on pregnancy risk and dropout rates and to examine the socio-demographic characteristics of users. Methods: This cross-sectional study with a retrospective cohort approach used calendar data from the 2017 Indonesia Demographic and Health Survey (IDHS). The study population included all contraceptive use episodes within five years, resulting in 46,461 episodes. Data were obtained from the 2017 IDHS socio-demographic questionnaire and contraceptive calendar. Multivariate logistic regression was used for analysis. Results: There was no significant difference in dropout rates between traditional and modern contraceptive users (OR = 0.92; 95% CI = 0.79–1.05). However, users of traditional methods had a 2.46-times higher risk of pregnancy compared with modern method users (95% CI = 2.19–2.76). Traditional contraceptive use was more common among women aged 40–49 years, with parity ≥ 5, higher education, and higher economic status, living in urban areas, and residing in Sumatra, Kalimantan, and Sulawesi (*p* = 0.001). Conclusions: Traditional contraceptive use is associated with a higher risk of pregnancy. Strengthening counseling services through comprehensive, pluralistic, and participatory approaches is essential while respecting individuals’ rights in contraceptive choice.

## 1. Introduction

The initial philosophy of the family planning (FP) program was a solution for population control [1]. Since then, contraceptive methods with high rates of effectiveness in preventing pregnancy have been developed, which we know as modern contraception [2,3]. Short-term methods (pills, injections, condoms, vaginal barriers), long-term methods (implants and IUDs), and permanent/sterilization methods (tubal ligation/vasectomy) are various types of modern contraception recommended by the World Health Organization (WHO) [4]. Globally, the use of modern contraception increased from 35% in 1990 to 45% in 2021. The proportion of users of short-term (46%) and long-term/permanent (44%) methods is not too different [5]. Indonesia is one of the countries that has successfully implemented family planning programs, reaching its peak in the 1970s–1990s [6]. This can be seen in the Total Fertility Rate (TFR), which dropped significantly from 5.61 in 1970 to 3.33 in 1990. After this, it stagnated between 2000 and 2020 at 2.34–2.18 [7]. Contraceptive use in Indonesia is quite different from global levels. Although cumulatively it appears to have increased from 49.7% to 63.6% in three decades [8], the trend in its use has tended to remain stable from the 2000s to 2017 [8,9].

In addition to stagnation, there are other issues that need to be considered in the dynamics of contraceptive use in Indonesia. The results of the 2017 Indonesian Demographic and Health Survey (IDHS) state that around 34% of women who use contraceptive methods decide to stop using them before one year of use or drop out [8]. This is a fairly high figure, similar to that in other countries such as Oman [10] and Ethiopia [11]. The most commonly reported reason was due to side effects or health issues (33.2%) [10]. Based on contraceptive method, the three highest dropout rates in Indonesia were for the pill (46%), injections (28%), and male condoms (27%) [8]. A similar situation was found in a study in the United States, where the highest dropout rates were for barrier methods and the pill [11].

Discontinuation of contraceptive use is associated with poor quality of counseling services [12]. In Indonesia, the quality of counseling is measured by the Method Information Index (MII), and in reality, the MII score is still low [8]. Counseling is one of the basic foundations of human rights-based contraceptive services, ensuring that contraceptive use is a free and informed choice [5]. Family planning providers must provide open counseling on all contraceptive methods, without any tendency to direct or impose a particular method [13], so that contraceptive use will better ensure user satisfaction and prevent discontinuation or switching to other less effective methods, such as traditional contraception [2,3]. In this study, traditional contraceptive methods refer specifically to periodic abstinence (rhythm method) and withdrawal (coitus interruptus), consistent with the Demographic and Health Survey classification.

In contrast to the global increase in the use of modern contraceptives, the use of traditional contraceptives has declined from 6% to 5% over the last three decades [5]. However, some countries have experienced different trends, such as Uttar Pradesh in India [14] and Indonesia [8]. In Indonesia, the number of traditional contraceptive users has increased, and in 2017, the number exceeded the global figure of 6% [8,15]. This anomaly is not only in terms of the increase in numbers but also in terms of who the users are. Traditional contraception in Indonesia is more commonly found among users with higher education levels, living in urban areas, and with higher socioeconomic status [8]. Similar conditions are also beginning to be found in several other countries, such as India, Jordan, and Iran [14,16,17]. While the lower rates of effectiveness of traditional methods are established globally, the simultaneous increase in traditional use, stagnation of modern use, and high discontinuation rates in Indonesia create a unique policy dilemma. This study provides the first direct comparison of pregnancy risk and discontinuation among Indonesian women using calendar data, and identifies the specific sociodemographic groups driving traditional method use particularly among educated, urban, higher-income women. These findings offer evidence to inform rights-based family planning counseling that respects reproductive autonomy while ensuring informed choice.

Indonesia is one of the largest and most populous countries in the world, with more than 270 million inhabitants distributed across thousands of islands and characterized by substantial cultural, social, and economic diversity. These contextual factors play an important role in shaping reproductive health behaviors, including contraceptive preferences and practices. In addition, religion and cultural values are closely intertwined with family planning decisions, particularly in a predominantly Muslim population. Contraceptive use should not only be viewed from a demographic perspective aimed at reducing fertility rates, but also as an essential component of reproductive rights and personal autonomy. The ability to choose a contraceptive method allows individuals and couples to improve their quality of life, plan their families according to their preferences, and enhance sexual well-being. Therefore, understanding contraceptive choice requires consideration of both effectiveness and individual preferences.

In this context, traditional contraceptive methods such as withdrawal are also influenced by cultural and religious perspectives. In Islamic teachings, the practice of ‘azl (i.e., coitus interruptus or withdrawal) has historically been discussed and is generally considered permissible under certain conditions, particularly when it is mutually agreed upon by both partners. This perspective contributes to the continued use of traditional methods in some populations.

Traditional contraception is usually perceived as old-fashioned contraception, used by the less educated, lower socioeconomic status, and rural communities. The phenomenon of increased use of traditional contraception, especially among the “privileged” segment of users, raises questions about whether this is a cause for concern and what the implications are for family planning programs. This study aims to analyze the effectiveness of contraception based on the risk of pregnancy and the dropout rate, as well as to examine the significance of the sociodemographic characteristics of modern and traditional contraceptive users in Indonesia.

## 2. Materials and Methods

### 2.1. Study Design

This quantitative study uses 2017 IDHS data through two approaches, namely cross-sectional to compare sociodemographic characteristics, and retrospective cohort through the calendar data section to analyze dropout and pregnancy risk during contraceptive use for 5 years prior to the 2017 IDHS.

### 2.2. Population and Sample

The study population consisted of all episodes of contraceptive use in the 2017 IDHS calendar data, totaling 58,047 episodes. A total of 46,461 episodes of contraceptive use among married and ever-married women were identified as the study sample for pregnancy risk analysis, then narrowed down to 26,762 total episodes of contraceptive use over 5 years for dropout analysis.

**Figure A1:**
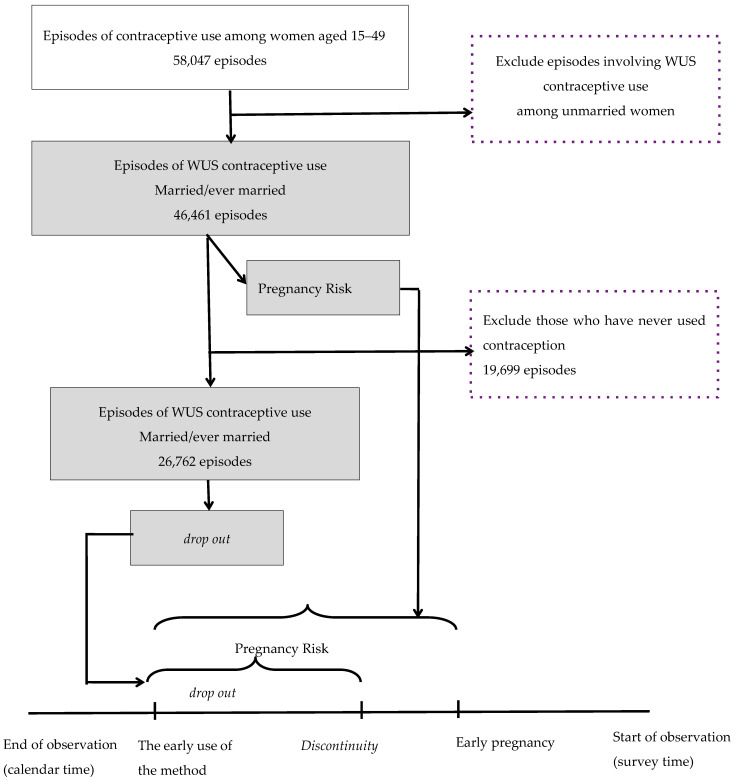
Flow diagram of sample selection from the 2017 IDHS.

Inclusion criteria for the pregnancy risk analysis were: (1) married or ever-married women aged 15–49 years; (2) having at least one contraceptive use episode recorded in the calendar during the five years prior to the 2017 IDHS; and (3) complete data on sociodemographic variables. Exclusion criteria were: (1) episodes with missing contraceptive method information; (2) episodes with missing pregnancy outcome data; and (3) episodes where the contraceptive use duration was less than one month. Non-users (women who never used any contraceptive method during the observation period) were included as a separate comparison group to assess the relative effectiveness of contraceptive methods against no method use. For dropout analysis, we additionally required episodes with a known discontinuation status and a minimum observation period of one month. A flow diagram of the sample selection process is presented in Figure A1 (See Appendix A).

### 2.3. Measurement

The data used was from the 2017 IDHS, which employed a questionnaire on social and demographic characteristics and a calendar containing a history of contraceptive method use by married women during the five years prior to the 2017 IDHS.

### 2.4. Data Collection

The focus of observation on calendar data in this study was the history of contraceptive types, duration of use, and pregnancy events during the observation period. The types of contraception used in this study were classified into three groups: (1) modern contraception in the form of sterilization, IUDs, implants, injections, pills, and condoms; (2) traditional contraceptive methods defined as periodic abstinence (rhythm method) and withdrawal (coitus interruptus), based on the Demographic and Health Survey (DHS) classification—these are the only methods categorized as traditional in the dataset used; (3) non-users of contraception, namely women who never used any form of contraception during the observation period. Meanwhile, the focus of observation on socio-demographic characteristic data was only on data on marital status, age, parity, education, economic level, place of residence, and regional area.

Pregnancy risk was measured based on the occurrence of a pregnancy event during an episode of contraceptive use, as recorded in the contraceptive calendar. For each contraceptive use episode, we identified whether a pregnancy occurred while the woman was using a given method. The outcome was binary: pregnant (uncensored) or not pregnant (censored) during the observation period. Pregnancy risk is expressed as the odds of becoming pregnant while using a specific contraceptive method compared to a reference method (IUD), after adjusting for sociodemographic factors and censoring. The censoring process accounted for episodes where women switched methods, discontinued use, or reached the end of the observation period without experiencing pregnancy.

In addition to measuring dropouts and pregnancy rates, a sensor process was used as a control variable in the analysis. The sensors used included dropout sensors, switch sensors, and observation duration sensors (calendar time). The sensor process revealed that the number of dropout sensors was 83.03%, which means that the dropout rate was 16.97%. Meanwhile, for pregnancy events, the condition of not being pregnant (censored) was identified at 85.47%, so that the observed pregnancy event was 14.53% (Table 1). Due to the large number of censored outcome variables, the analysis results were treated as index values rather than actual (absolute) values.

### 2.5. Data Analysis

Identification of differences in sociodemographic characteristics among contraceptive users was tested using bivariate logistic regression analysis and multivariate logistic regression analysis to estimate dropout rates and pregnancy risk. Results were interpreted using odds ratio (OR), 95% confidence interval, and statistical significance *p* < 0.05.

## 3. Results

### 3.1. Characteristics of Contraceptive Users

Table 2 shows that awareness of family planning is quite good, with a strong preference for more effective modern methods. However, the large proportion of non-users (42%) remains an important focus for family planning programs.

Based on marital status, contraceptive users (both modern and traditional) are more commonly found among married women, while non-users are predominantly divorced women. In terms of age, modern contraceptive use peaks among those aged 20–29 and then declines sharply, while traditional methods are most commonly used among those aged 30–39, and non-users surge among those aged 40–49. Based on parity, the use of modern methods tends to decline as the number of children increases, while non-users increase with higher parity, and traditional methods are most often chosen by women who do not yet have children. In terms of education, users of traditional contraception are mostly found among high school and college graduates, modern methods are dominant among junior high school graduates/high school dropouts, and non-users are highest among the low-educated group. Geographically, the use of modern methods is higher in rural areas, while traditional methods and non-users are more prevalent in urban areas. Regionally, Sumatra, Kalimantan, and Sulawesi have the highest proportion of contraceptive users (modern and traditional), while NTB, NTT, Maluku, and Papua are dominated by non-users.

### 3.2. Comparison of Characteristics of Contraceptive Users

A comparison of characteristics between users and modern contraception as a comparison was conducted, as shown in Table 3.

Based on the results of multinomial logistic regression analysis using modern contraceptive users as the comparison group, there are two significantly different socio-demographic patterns between traditional contraceptive users and non-users.

First, the tendency to use traditional methods compared to modern methods is significantly higher among women with the following characteristics: older age (peaking in the 40–49 age group), high parity (especially among women with five children), higher education and economic levels (especially high school/university graduates and the upper economic group), and residence in urban areas and in the regions of Sumatra, Kalimantan, and Sulawesi. Second, the tendency not to use contraception at all (non-users) is significantly associated with divorced marital status, age of 40–49 years, parity of more than one child, higher education, and residence in urban areas and Eastern Indonesia (NTB, NTT, Maluku, Papua). The upper economic group also showed a tendency to be non-users, although the middle economic group preferred modern methods. These results indicate that although factors such as older age, high parity, and urban location increase the likelihood of using traditional methods or being non-users, higher education and economic status actually encourage the use of traditional methods or even no contraception at all. This means that these two factors do not always drive the adoption of modern methods, as is often assumed.

### 3.3. Contraceptive Effectiveness

**Figure 1:**
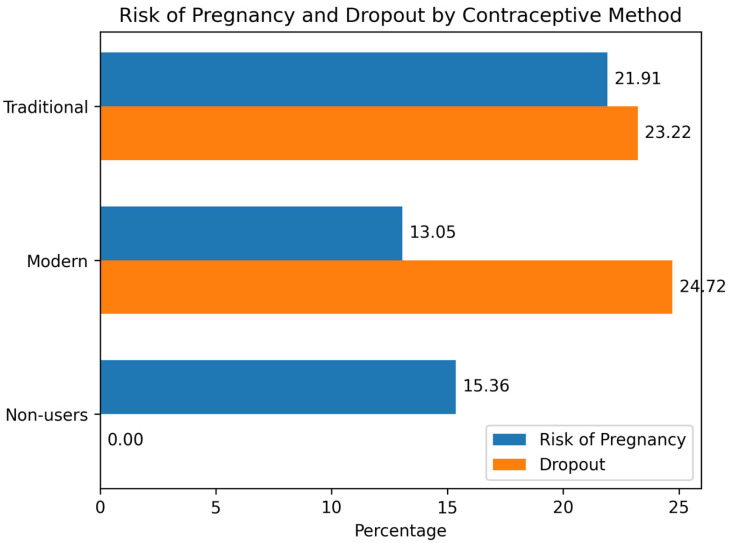
Percentage of contraceptive effectiveness based on dropout and pregnancy rates.

Figure 1 shows that contraceptive effectiveness can be assessed from two aspects: the risk of pregnancy and the rate of discontinuation (dropout). The results of the study show that the highest risk of pregnancy is experienced by users of traditional contraception (21.91%), followed by users of modern methods (13.05%), while non-users have a risk of 15.36%. This indicates that in preventing pregnancy, modern methods are still more effective than traditional methods, although both methods are still better than not using contraception at all. However, in terms of sustainability of use, both methods face serious challenges. The dropout rate for modern methods reached 24.72%, slightly higher than traditional methods (23.22%). This figure shows that nearly a quarter of users of both methods are likely to discontinue use within the observed period. To further examine these patterns, Table 4 presents odds ratios (OR) and 95% confidence intervals (CI) comparing contraceptive effectiveness by method type.

The multivariate logistic regression analysis in Table 4 shows that traditional contraceptive users had an estimated 8% lower tendency to discontinue contraceptive use within 12 months compared to modern method users, but this tendency was not statistically significant (OR = 0.92). This indicates that both types of contraceptives actually have similar dropout tendencies. In contrast to dropout rates, the analysis of pregnancy occurrence during contraceptive use found that women using traditional methods had a significantly 2.46 times higher likelihood of becoming pregnant (OR = 2.46) compared to modern contraceptive users.

Although dropout rates did not differ significantly by contraceptive group, significant differences were observed among individual contraceptive methods. MOP and MOW had perfect continuation rates due to their permanent nature (no discontinuation). Implant users had a 68.8% lower risk of discontinuing use compared to IUD users. Meanwhile, other methods such as pills, injections, and traditional methods had significantly higher dropout risks than IUDs, 3.2 times higher for pill users, while injection and traditional method users both had 1.5 times higher risk. Condom users also had a higher dropout risk (1.2 times) than IUD users, but this was not statistically significant.

Regarding dropout by contraceptive group, traditional methods tended to have a higher dropout risk but not significantly so, whereas the detailed analysis showed that long-acting contraceptive methods were the most protective against dropout. However, for pregnancy occurrence, there was consistency in the results both by contraceptive group and by individual method type. MOP and MOW users had a 95.1% lower risk of becoming pregnant compared to IUD users as the reference. This is consistent with their permanent nature. Implants did not differ significantly from IUDs in terms of pregnancy risk, meaning that implant effectiveness is equivalent to IUDs in preventing pregnancy. In contrast to MOP/MOW and implants, other methods had significantly higher risks of pregnancy compared to IUDs. Injections carried nearly 2 times the risk of pregnancy, pills 5 times, condoms 4 times, and traditional methods 6 times higher. In other words, traditional contraceptives had the highest risk of pregnancy among all contraceptive methods.

### 3.4. Socio-Demographic Determinants of Contraceptive Discontinuation (Dropout) and Risk of Pregnancy

Table 5 presents The multivariate analysis reveals distinct socio-demographic profiles for contraceptive dropout and pregnancy risk, indicating that these outcomes are driven by different mechanisms, with age playing a central role through the lens of climacteric fecundity. Climacteric fecundity, the natural decline in fertility associated with aging particularly from the late 30 s onward, significantly shapes both outcomes. For pregnancy risk, the protective effect of age was striking. Compared to adolescents aged 15 to 19 years, women aged 30 to 39 years had a 20% lower risk (aOR = 0.80), and those aged 40 to 49 years had an 80% lower risk (aOR = 0.20). This steep gradient reflects the biological reality of declining ovarian reserve, reduced oocyte quality, and lower fecundability as women approach and traverse the climacteric period. In contrast, for contraceptive dropout, women aged 20 to 29 years (aOR = 0.71) and 30 to 39 years (aOR = 0.82) were less likely to discontinue than adolescents, while those aged 40 to 49 years showed no significant difference from the youngest group (aOR = 1.16, *p* > 0.05). This suggests that climacteric related concerns, such as irregular bleeding, side effect intolerance, or the perception that pregnancy is no longer possible, may drive older women to discontinue contraception unnecessarily, exposing them to residual pregnancy risk despite reduced fecundity.

Beyond age, distinct patterns emerged for discontinuation. Women with two or three children had higher dropout likelihood (aOR = 1.48 and 1.84, respectively), suggesting that achieving desired family size may reduce motivation for consistent method use. Higher education also increased dropout risk, with high school or college graduates showing an aOR of 1.29, potentially reflecting greater awareness of side effects or preference for nonhormonal methods. Geographically, women in Sumatra, Kalimantan, and Sulawesi were more prone to discontinuation (aOR = 1.19), while those in eastern Indonesia (Nusa Tenggara, Maluku, Papua) were significantly less likely to drop out (aOR = 0.77), pointing to regional variations in service quality and cultural acceptance. Divorce, economic status beyond the lowest quintile, and urban rural residence were not significant predictors of dropout after adjustment.

For pregnancy risk, the strongest predictors were geographic and educational. Residing in eastern Indonesia elevated risk substantially (aOR = 2.15), followed by higher education (aOR = 1.40 for high school or college graduates). These groups may rely more on less effective traditional methods or experience contraceptive gaps despite not desiring pregnancy. Protective factors against pregnancy risk included divorce (aOR = 0.80), high parity (parity 2: aOR = 0.30; parity 3: aOR = 0.26), and higher economic status (bottom quintile: aOR = 0.85; medium quintile: aOR = 0.71). Rural residence slightly reduced pregnancy risk (aOR = 0.88), possibly due to a mix of different methods or lower sexual exposure. Notably, the protective effect of higher economic status against pregnancy risk contrasts with its null effect on dropout, suggesting that wealthier women who discontinue can still avoid pregnancy through better access to emergency contraception or private services.

To complement the Indonesian findings, a comparative narrative analysis was conducted across eight countries (Appendix A Table A1)**,** covering regions in the Middle East, Southeast Asia, and Sub-Saharan Africa. The comparison reveals substantial variation in both the prevalence and patterns of contraceptive use. Across countries, the prevalence of modern contraceptive use ranges from 30.7% in Ghana to 55% in Iran, while traditional method use remains notable, reaching as high as over 64% among postpartum women in the Democratic Republic of Congo. In several countries, such as Nigeria and Iran, traditional methods still constitute a considerable proportion of contraceptive use, indicating that modernization does not uniformly reduce reliance on traditional practices.

The characteristics of traditional method users vary across contexts. In countries such as the Philippines, Nigeria, and Iran, traditional methods are more commonly used by women with higher education levels, urban residence, and higher socioeconomic status. In contrast, in settings like Jordan, traditional method use is more associated with younger age, higher parity, and rural residence. These differences suggest that the socio-demographic profile of traditional method users is highly context-specific. Despite these variations, several consistent patterns emerge. Fear of side effects of modern contraception is the most frequently reported driver across nearly all countries, including the Philippines, Nigeria, Iran, Ghana, Ethiopia, and the Democratic Republic of Congo. Other commonly reported factors include husband opposition, religious beliefs, lack of knowledge, and perceived convenience of traditional methods.

Additionally, knowledge of the fertile period appears to play an important role in certain contexts, such as in the Philippines, where correct knowledge is significantly associated with increased use of rhythm methods. However, this factor is not consistently measured across countries. The role of education, economic status, and residence also shows mixed patterns. Higher education is associated with increased use of traditional methods in several countries, while economic status and residence demonstrate inconsistent associations across settings. Urban residence, however, tends to be linked with higher use of traditional methods in multiple countries. Overall, the comparative analysis highlights both divergent national patterns and shared underlying drivers, particularly related to perceptions of modern contraception and socio-cultural influences.

## 4. Discussion

### 4.1. Contraceptive Use

The study results confirm the national pattern in which the majority of women (58%) are contraceptive users, with modern methods dominating (52%) compared to traditional methods (6%) [8]. Although the proportion of traditional users appears small, the figure has increased from 4% (2012) [15] to 6% (2017) [8], exceeding the global average (5%), even though global data generally shows a decline [5]. The regions of Northern Africa and Western Asia are the largest users of traditional contraception at 15% [5]. A country experiencing a similar situation is Uttar Pradesh, India [14]. The anomaly occurring in Indonesia is important to note, where the effectiveness of traditional contraception is lower than modern methods and has the potential to increase the risk of unwanted pregnancies [18].

The proportion of traditional contraceptive users observed in this study (6%) is slightly higher than previous estimates (approximately 4%). However, this difference should be interpreted with caution. The present study was not designed to statistically test differences in prevalence between studies, and variations may be influenced by differences in study design, sampling, time period, and measurement approaches. Furthermore, although the absolute difference appears small, it may still reflect a consistent pattern of sustained use of traditional methods over time. Future studies with comparable designs and sufficient statistical power are needed to assess trends in prevalence more robustly.

### 4.2. Contraceptive Effectiveness: Risk of Pregnancy vs. Dropouts

Based on effectiveness analysis, although the dropout rate for traditional contraception is not significantly different from modern methods in general, the risk of causing pregnancy is actually the highest. This contrasts with Long-Acting Reversible Contraceptives (LARCs) such as IUDs, implants, and sterilization, which show the highest effectiveness in preventing pregnancy. In line with [2,3,4]. LARCs not only have very high rates of effectiveness in preventing pregnancy, even with imperfect use [2,3], but also offer high continuity of use due to their long-term and reversible nature (except sterilization) [4].

Conversely, Short-Acting Reversible Contraception (SARC) such as pills and injections, although classified as highly effective when used consistently and correctly (perfect use) [2,3], is the largest contributors to dropout rates [8,11]. This vulnerability is due to a drastic decrease in effectiveness with imperfect daily use (common use) [2,3] and high rates of side effect complaints [8,10]. The 2017 IDHS data confirms this by reporting that 46% of contraceptive discontinuation was due to pills and 28% due to injections, primarily because of discomfort from side effects or health problems (33%) [8]. Interestingly, traditional contraceptives such as periodic abstinence and withdrawal were not significant predictors of dropout. This is explained by the profile of their users, who are predominantly women with higher education living in urban areas, thus having better understanding and confidence in maintaining their health [19] through consistency and accuracy in using traditional methods, which in turn encourages continuity.

Unlike previous studies that rely on published effectiveness rates from clinical trials or global averages, our pregnancy risk estimates are calculated directly from reported pregnancies during contraceptive use episodes in the IDHS calendar. This provides a real-world, typical-use estimate specific to the Indonesian context. The finding that traditional method users have 2.46 times higher odds of pregnancy compared to modern method users is derived directly from the reported experiences of 46,461 contraceptive episodes in Indonesia between 2012 and 2017, not extrapolated from WHO or other international sources.

### 4.3. Socio-Demographic Characteristics of Contraceptive Users

In fact, the use of modern contraception is considered a barometer of the success of rational and ideal family planning behavior, while traditional methods are often seen as a second or even last choice by users and providers. However, the results of this study are contrary to this perspective. By using modern users as a comparison, sociodemographic characteristics were identified that significantly made users prefer traditional methods.

The study revealed that there is no significant difference in the tendency to choose traditional or modern contraceptive methods based on marital status. These findings indicate that marital status alone is not a sufficient predictor of contraceptive choice, which is instead determined more by individual rights [20], sexual satisfaction [21], and fear of side effects [14,22,23,24].

Research also confirms that age significantly influences contraceptive choice, with a stronger tendency to use traditional methods among older women. This is primarily driven by concerns about the side effects of modern methods [14,22,23,24] and perceptions of a natural decline in fertility [25,26]. Although the risk of pregnancy does decrease with age, it is important to continue providing evidence-based counseling to prevent unwanted pregnancies that pose a high risk to health.

Furthermore, the results of this study show that parity significantly influences contraceptive choice, with women who have five children being 1.5 times more likely to choose traditional methods. This finding is consistent with other studies [16,27]. This is driven by long reproductive experience, including bad experiences with modern contraception or beliefs about the safety of traditional methods that have been proven not to fail before [14,23]. Additionally, the perception that fertility has declined [25,26] or that the number of children [16,27] is sufficient makes traditional methods seem adequate, supported by cultural factors such as the belief that “many children bring much fortune” [28] and the flexibility to stop using them at any time without a period of fertility adjustment [4].

The level of education of women in this study significantly influenced their preference for traditional contraception. The analysis results found a tendency to use traditional contraception among educated groups [5,27,29,30]. Awareness of natural methods and minimal intervention can increase autonomy in decision-making among women with secondary to higher education [19]. They consciously consider the advantages of natural methods that involve minimal medical intervention and side effects, even though they are less effective. In addition, cultural values, religiosity, and personal beliefs [31], such as the application of the principle of ‘azl **(i.e., coitus interruptus)** in Islam, which allows for natural pregnancy regulation, also shapes this choice [32]. Thus, increased education and empathetic information delivery, as well as the involvement of educational institutions and professionals, are necessary to effectively reach this group.

A high economic level is significantly associated with the tendency to use traditional contraception, a finding consistent with various other studies [5,17,27,29]. This indicates that contraceptive choice is not solely determined by the financial ability to access modern methods. The main factor at play is knowledge about the fertile period [29,33], which can increase confidence and independence in applying natural methods such as the calendar or cervical mucus observation. Additionally, concerns about the side effects of modern methods [14,22,23,24], supported by the myth that contraceptives can “move” within the body [34,35], can be a strong barrier, even when cost is no longer an issue. Therefore, health promotion efforts need to strengthen educational and evidence-based communication to convey the higher risk of failure associated with traditional contraception, thereby encouraging safer and more effective choices in line with existing economic capabilities.

Place of residence is a significant predictor in the choice of traditional contraception [5,16,17,23,27,29]. Women in urban areas are more likely to choose this method, driven by natural lifestyle trends [29] and better access to information [36]. Regionally, usage is higher in Sumatra, Kalimantan, and Sulawesi, which is associated with the slower introduction of family planning programs compared to Java–Bali [6] and the influence of matrilineal and patrilineal cultures in some Sumatran tribes that have a preference for certain genders and value flexibility in pregnancy planning [37].

To contextualize our findings within the broader global landscape, we compared our results with existing studies from other countries (Table A1). Despite varying cultural and health system contexts, several convergent patterns emerge across these settings. First, traditional contraceptive use in Indonesia is consistently associated with higher education and urban residence [10]. This result is consistent with other research findings, that is found that women of childbearing age who are 35 years or older, have a higher education level, and live in urban areas tend to use traditional contraceptive methods [38]. Furthermore, women with good knowledge about family planning methods and their fertile period also tend to use traditional contraceptive methods [10]. A similar pattern is seen in countries such as the Philippines, where traditional contraception remains popular among educated and older age groups, supported by factors such as limited program priorities, access constraints, and religious norms [33].

**Other research in Indonesia** documented challenges in male contraceptive uptake in Indonesia, highlighting that traditional gender roles and social norms influence family planning decisions [39]. Similarly, sterilization adoption in Indonesia remains low (only 3.8% of couples) with significant gender disparity (female: 4.5%; male: 0.1%), demonstrating how social norms and traditional gender roles, including the belief that contraception is a woman’s responsibility, continue to shape family planning practices [3].

These converging patterns suggest that the Indonesian situation is not entirely unique but rather reflects broader challenges in rights-based family planning counseling that are shared globally. However, Indonesia remains distinctive in two important respects. First, the 2017 IDHS shows that 53.95% of married women were using modern contraceptive methods, and 6.64% traditional methods, while 39.42% were not using any method [1]. Notably, traditional method use increased from 4% to 6% between 2012 and 2017, while global trends generally show a decline in traditional method use as modernization progresses. Second, the profile of traditional method users in Indonesia (educated, urban, higher-income women) contrasts with patterns observed in Sub-Saharan Africa, where traditional use is typically associated with lower education and rural residence [23].

This distinctive profile suggests that in Indonesia, traditional method use is not merely a matter of limited access or lack of information, but rather an active, informed choice among women who might otherwise be expected to prefer modern methods. The comparative analysis reinforces our central argument: improving contraceptive effectiveness in Indonesia requires not just increasing access to modern methods, but fundamentally strengthening rights-based counseling that respects women’s reproductive autonomy while ensuring they have complete and accurate information about the relative effectiveness of different methods.

### 4.4. Socio-Demographic Determinants of Contraceptive Discontinuation and Risk of Pregnancy

Contraceptive dropout rates are higher among older women, those with 2–3 children, those with higher education, and those with higher socioeconomic status, and in the regions of Sumatra, Kalimantan, and Sulawesi. Among older women, natural decline in fertility [40] and concerns about hormonal side effects [5,14,27] are the primary causes. Having 2–3 children is associated with the ideal target of three children in Indonesia [8]. Higher socioeconomic status, however, leads to inconsistent use due to dissatisfaction with the contraceptive method used [41]. Contraceptive users with higher education are more aware of the risks of side effects and thus tend to switch to safer methods [22]. Geographical challenges in eastern regions hinder access to family planning services [42].

The risk of pregnancy is higher among highly educated women, those living in urban areas, and those outside Java–Bali. Cultural acculturation in Java–Bali, as a metropolitan region, also influences contraceptive use patterns that are vulnerable to the risk of unintended pregnancy. Conversely, divorced women, older women, those with high parity, and those with higher socioeconomic status actually face lower risks. Educated groups [5,27,29,30] and urban populations [5,16,17,23,27,29] are more likely to use traditional contraceptives with low effectiveness and experience periods without contraception even when they do not wish to have more children.

### 4.5. Implications for Family Planning Programs

Research findings on the increase in traditional contraceptive users, which are dominated by educated, high socioeconomic status urban women, amid stagnant modern user numbers and high discontinuity, have fundamental implications for family planning counseling services.

The first implication is the need to guarantee the right to comprehensive information; counselors are required to provide information proportionally on all contraceptive methods, both modern and traditional. Second, counseling must respect the right to autonomy according to the client’s needs, not impose a particular method. While respecting the client’s decision to choose a traditional method, it is the counselor’s duty to ensure that the client understands how to use it correctly. Third, stagnation and discontinuity in modern contraception demand high-quality services that are responsive to managing complaints about side effects. Finally, these findings can serve as a basis for expanding success indicators beyond modern contraceptive coverage rates to include service quality and client satisfaction. Thus, increased use of traditional contraceptives among certain groups should be seen as a signal for the need for more pluralistic, participatory counseling services that respect the human rights of every individual in determining their reproductive decisions.

### 4.6. Strength and Study Limitation

This study uses 2017 IDHS data, which is the most recent nationally representative survey with detailed contraceptive calendars. However, contraceptive use patterns may have changed since 2017. The COVID-19 pandemic may have affected access to family planning services and contraceptive use patterns. Future research using more recent data (e.g., 2022 or upcoming 2027 IDHS) is needed to confirm trends. The findings should therefore be interpreted as reflecting patterns from the 2012–2017 period. Moreover, this study did not examine interaction effects between sociodemographic variables (e.g., education and residence, age and parity). Future studies should explore potential effect modifications to better understand the complex determinants of traditional contraceptive choice.

The sample size for traditional method users (n = 2665) was substantially smaller than for modern users (n = 24,097) and non-users (n = 19,699). This imbalance may affect the precision of estimates and could lead to overestimation of associations in bivariate and multivariate models. However, our use of robust confidence intervals and the large absolute number of traditional users (2665 episodes) still provide sufficient statistical power for the primary analyses. Nevertheless, results for traditional methods should be interpreted with caution, and replication in larger samples or pooled multi-survey data is warranted.

In this study, we did not adjust for menopausal status or age-related fertility decline. Women aged 40–49 may have reduced fecundity, which could affect both contraceptive choice and pregnancy risk. Future studies should incorporate biological markers of fecundity or conduct stratified analyses excluding perimenopausal women to isolate contraceptive effects from natural infertility.

The 2017 IDHS data do not include information on the frequency or regularity of sexual activity. Marital status was used as a proxy, but being married or unmarried does not necessarily indicate the presence or absence of regular sexual activity, which directly affects pregnancy risk. Future studies should incorporate measures of coital frequency or sexual activity patterns to more accurately estimate contraceptive effectiveness. Second, the data are from 2017, and patterns may have changed since then, particularly following the COVID-19 pandemic. Third, we did not adjust for menopausal status in women aged 40–49, as this information was not available in the dataset. Fourth, the sample size for traditional method users (n = 2665) was substantially smaller than for modern users (n = 24,097), which may affect the precision of estimates. Fifth, the DHS calendar data rely on self-reported information, which may be subject to recall bias, although the five-year recall period is standard in DHS methodology.

## 5. Conclusions

Although the overall dropout rate did not show a significant difference between traditional and modern contraception, traditional contraception carries a higher risk of pregnancy than modern contraception. This reinforces the fact that modern contraception is more effective than traditional contraception. Sociodemographic characteristics have been shown to not always protect against the tendency to use modern contraception. A more comprehensive transformation of counseling services for all contraceptive methods, which respects the diverse characteristics of users and involves the active participation of women and their husbands, is important to develop. Exploratory studies to examine the phenomenon of traditional contraceptive choice among “privileged” groups are important to obtain a complete picture of traditional contraceptive choice patterns in Indonesia.

## Figures and Tables

**Table 1:** Number censored episodes in the identification of dropouts and risk of pregnancy.

Outcomes	Total Episodes	∑Uncensored	∑Censored
Dropouts	26.762	4540	22.222
Risk of pregnancy	46.461	6752	39.709

**Table 2:** Frequency distribution of socio-demographic characteristics of contraceptive users in 2017 IDHS.

Variable	*n*	Contraceptive Method (%)		
		Traditional	Modern	Non-Users
	46,462	*n* = 2665	*n* = 24,097	*n* = 19,699
Marital Status				
Married	43,989	5.97	53.72	40.30
Ever married	2472	1.55	18.75	79.70
Age				
15–19	835	1.99	53.21	44.81
20–29	12,181	6.65	68.91	24.44
30–39	18,531	6.8	59.28	33.92
40–49	14,914	3.88	28.65	67.47
Parity				
0	10,889	7.33	59.84	32.84
1	12,079	5.82	61.34	32.84
2	12,490	4.92	47.94	47.14
3	6446	5.21	41.56	53.23
4	2615	4.45	37.63	57.93
5	1942	5.01	26.86	68.12
Education				
No schooling/did not complete nine years of schooling	15,825	2.7	45.78	51.53
Completed junior high school/did not complete senior high school	12,277	4.64	58.59	36.77
High school graduate	18,359	9.09	52.62	38.29
Socioeconomic Status				
Lowest	8048	4.01	52.32	43.68
Bottom	9213	4.34	55.16	40.5
Middle	9749	5	54.41	40.6
High	9882	6.76	50.85	42.4
Highest	9569	8.24	46.77	44.99
Place of Residence				
Urban	22,682	6.88	50.17	42.95
Rural	23,779	4.65	53.48	41.87
Province				
Java–Bali	27,797	5.1	51.96	42.94
Sumatra, Kalimantan, Sulawesi	16,021	7.15	52.84	40.01
West Nusa Tenggara, East Nusa Tenggara, Maluku, Papua	2643	3.84	44.95	51.21

**Table 3:** Comparison of characteristics of traditional contraceptive users and non-users versus modern users.

Variable	Traditional vs. Modern OR (95% CI)	Non-Users vs. Modern OR (95% CI)
Age		
15–19	Ref	Ref
20–29	2.59 (1.58–4.25) ***	0.42 (0.36–0.49) ***
30–39	3.07 (1.88–5.04) ***	0.68 (0.59–0.78) ***
40–49	3.63 (2.20–5.97) ***	2.80 (2.43–3.22) ***
Parity		
0	Ref	Ref
1	0.77 (0.70–0.86) ***	0.98 (0.92–1.03)
2	0.84 (0.75–0.94) **	1.79 (1.70–1.89) ***
3	1.02 (0.89–1.17)	2.33 (2.19–2.49) ***
4	0.97 (0.79–1.19)	2.81 (2.56–3.07) ***
5	1.52 (1.21–1.92) ***	4.62 (4.14–5.15) ***
Education		
Did not complete junior high school	Ref	Ref
Junior high school graduate/high school/vocational school dropout	1.34 (1.18–1.53) ***	0.56 (0.53–0.59) ***
High school/vocational school graduate/college graduate	2.93 (2.62–3.27) ***	0.65 (0.62–0.68) ***
Socioeconomic status		
1	Ref	Ref
2	1.03 (0.88–1.20)	0.88 (0.83–0.94) ***
3	1.20 (1.04–1.39) *	0.89 (0.84–0.95) ***
4	1.73 (1.51–1.99)***	1.00 (0.94–1.06)
5	2.30 (2.01–2.64) ***	1.15 (1.08–1.23) ***
Place of Residence		
Urban	Ref	Ref
Rural	0.63 (0.58–0.69) ***	0.91 (0.88–0.95) ***
Province		
Java and Bali	Ref	Ref
Sumatra, Kalimantan, Sulawesi	1.38 (1.27–1.50) ***	0.92 (0.88–0.95) ***
West Nusa Tenggara, East Nusa Tenggara, Maluku, Papua	0.87 (0.71–1.07)	1.38 (1.27–1.50) ***
**Marital Status**		
Married	Ref	Ref
Divorced/Deceased Spouse	0.74 (0.53–1.03)	5.66 (5.11–6.28) ***

*: *p* value <0.05,**: *p* value< 0.01, ***: *p* value <0.0001.

**Table 4:** Comparison of odds ratios (OR) and 95% confidence intervals (CI) for contraceptive effectiveness by type of contraceptive method in Indonesia (IDHS 2017).

Factors	Contraceptive Effectiveness			
	Drop out ^1^		Risk of Pregnancy ^2^	
	OR	95% CI	OR	95% CI
Contraceptive Group				
Modern	Ref	Ref	Ref	Ref
Traditional	0.92	0.79–1.05	2.46 ***	2.19–2.76
Type of Contraceptive Method				
Male/Female Sterilization/MOP/MOW	n/a	n/a	0.04 ***	0–0.31
IUD	Ref	Ref	Ref	Ref
Implant	0.31	0.17–0.55	1.02	0.74–1.39
Injection	1.58 *	1.03–2.41	1.95 ***	1.55–2.45
Pills	3.27 ***	2.12–5.03	5.05 ***	4.01–6.33
Condom	1.60	0.95–2.98	4.45 ***	3.34–5.92
Traditional	1.50	0.93–2.41	6.08 ***	4.76–7.76

^1^ Study population: women using contraception who want to limit childbearing, ^2^ Study population: married/ever-married women. * *p* value <0.05; *** *p* value < 0.001.

**Table 5:** Socio-demographic determinants of contraceptive discontinuation (dropout) and risk of pregnancy among Indonesian women: IDHS 2017.

Factor	Dropout ^1^		Risk of Pregnancy ^2^	
	OR (95% CI)	Adjusted OR (95% CI)	OR (95% CI)	Adjusted OR (95% CI)
Marital				
Married	Ref	Ref	Ref	Ref
Divorce	2.60 [2.17–3.10] ***	1.21 [0.98–1.49]	0.84 [0.74–0.95] *	0.80 (0.70–0.91) *
Age				
15–19	Ref	Ref	Ref	Ref
20–29	0.63 [0.51–0.77] ***	0.71 [0.60–0.82] ***	0.87 [0.74–1.02]	0.88 (0.74–1.06)
30–39	0.58 [0.48–0.71] ***	0.82 [0.68–0.97] *	0.57 [0.49–0.67] ***	0.80 (0.66–0.97) *
40–49	0.65 [0.53–0.79] ***	1.16 [0.91–1.47]	0.15 [0.12–0.17] ***	0.20 (0.16–0.25) *
Parity				
0	Ref	Ref	Ref	Ref
1	0.83 [0.77–0.89] ***	1.13 [1.01–1.26] *	0.59 [0.55–0.63] ***	0.52 (0.48–0.57) *
2	0.94 [0.87–1.01]	1.48 [1.29–1.69] ***	0.30 [0.28–0.33] ***	0.30 (0.27–0.33) *
3	1.11 [1.11–1.22] *	1.84 [1.54–2.17] ***	0.23 [0.20–0.25] ***	0.26 (0.23–0.30) *
4	0.70 [0.60–0.82] ***	0.98 [0.77–1.25]	0.24 [0.20–0.28] ***	0.32 (0.27–0.39) *
5	0.61 [0.49–0.75] ***	1.06 [0.77–1.46]	0.30 [0.25–0.35] ***	0.44 (0.37–0.54) *
Education				
Did not attend school/did not complete junior high school	Ref	Ref	Ref	Ref
Completed junior high school/did not complete high school	1.26 [1.17–1.36] ***	1.21 [1.08–1.36] **	1.54 [1.43–1.65] ***	1.17 (1.08–1.27) *
High school graduate/college graduate	1.39 [1.30–1.49] ***	1.29 [1.15–1.45] ***	1.91 [1.79–2.03] ***	1.40 (1.29–1.52) *
Economic Status				
Lowest	Ref	Ref	Ref	Ref
Bottom	1.22 [1.11–1.34] ***	1.26 [1.09–1.29] **	0.83 [0.76–0.90] ***	0.85 (0.77–0.93) *
Medium	1.22 [1.11–1.34] ***	1.13 [0.98–1.29]	0.72 [0.67–0.78] ***	0.71 (0.65–0.79) *
High	1.23 [1.12–1.35] ***	1.07 [0.92–1.24]	0.77 [0.71–0.83] ***	0.76 (0.69–0.84) *
Top	1.39 [1.27–1.52] ***	1.17 [0.99–1.37]	0.84 [0.78–0.91] ***	0.80 (0.72–0.90) *
Place of Residence				
Urban	Ref	Ref	Ref	Ref
Rural	0.99 [0.94–1.05]	0.99 [0.90–1.08]	0.97 [0.92–1.02]	0.88 (0.82–0.94) *
Province				
Java–Bali	Ref	Ref	Ref	Ref
Sumatra, Kalimantan, Sulawesi	1.30 [1.23–1.39] ***	1.19 [1.08–0.29] ***	1.35 [1.28–1.42] ***	1.42 (1.33–1.51) *
West Nusa Tenggara, East Nusa Tenggara, Maluku, Papua	0.75 [0.65–0.87] ***	0.77 [0.63–0.95] *	2.15 [1.95–2.36] ***	2.15 (1.92–2.41) *

^1^ Study population of female contraceptive users. *** *p*-value <0.001, ** *p*-value < 0.01, * *p*-value <0.05. ^2^ Study population of married/ever-married women.

## Data Availability

Publicly available datasets were analyzed in this study. These data are available from the Demographic and Health Surveys (DHS) Program at https://dhsprogram.com/ (accessed on 16 March 2026), subject to registration and approval of data access requests.

## Abbreviations

The following abbreviations are used in this manuscript:

IDHS	Indonesia Demography Health Survey
LAM	Lactational Amenorrhea Method
LARC	Long-Acting Reversible Contraception
MII	Method Information Index
SARC	Short-Acting Reversible Contraception
TFR	Total Fertility Rate
WHO	World Health Organization (WHO)

## Appendix A

**Table A1:** Comparative narrative analysis of traditional vs. modern contraceptive use across eight countries.

Aspect/Country	Jordan [16]	Philippines [33]	Nigeria [23]	Iran [17]	Ghana [23]	Uganda [23]	Ethiopia [23]	DR Congo [23]
**Prevalence of modern contraceptive use**	42.3% among married women (15–49 years)	38% among married women (2013)	43.9% ever used modern methods	55% using modern methods	30.7%	Not specified	Not specified	Not specified
**Prevalence of traditional contraceptive use**	18.9% (rhythm & coitus interruptus)	17% (rhythm 9.3%, withdrawal 22% in 2013)	32.4% (rhythm & withdrawal)	26.1% of contraceptive users (primarily withdrawal)	19.5%; abstinence higher among single young women	Withdrawal used by young adults	2.7% (withdrawal & breastfeeding)	>64% among postpartum women
**Characteristics of traditional method users**	Young age (15–24), high parity, living in southern & rural areas	Highly educated/college, urban, high income (rhythm); high school, not working, Catholic (withdrawal)	Highly educated, urban residence, high socioeconomic status	Age 15–24 years, university education, urban-born, childless, desire more children	Senior high school associated with abstinence; urban, educated	Women > 30 years, employed, living <30 km from town	Not detailed	Not detailed
**Main drivers of traditional method use**	Husband’s desire for more children, lack of access in south, cultural norms	Fear of modern side effects, ease of use, lack of access, religious beliefs	Fear of side effects (irregular menstruation, bleeding, weight change), negative peer experiences	Fear of modern side effects, comfort, husband’s opposition, belief in natural methods	Rhythm method most patronized; spousal communication, desire to space children	Not explicitly stated	Absence of side effects (70.1%), fear of hormonal contraceptives (53.8%), ease of use, no cost	Lack of knowledge, fear of side effects, religious considerations, husband opposition
**Knowledge of fertile period**	Not measured	Significant correlation (OR 1.58; correct knowledge → more rhythm use)	Not measured quantitatively	Not measured directly	Not measured	Not measured	Not measured	Not measured
**Role of education**	Bivariate significant, not significant in multivariate	Higher education → more rhythm use; secondary → more withdrawal use	Higher education does not always promote modern use	Higher education → more withdrawal use (OR 0.176 for low education vs. university)	Senior high school → more abstinence	Not significant	Not specified	Not specified
**Role of economic status**	Not significant	Lowest wealth quintile → more rhythm & withdrawal use	High economic status → higher contraceptive use, but many still use traditional	Not significant	Middle/rich categories → lower abstinence, higher met need	Richest women more likely to use traditional	Not specified	Not specified
**Role of residence**	Central & urban areas more likely to use modern	Urban areas more likely to use withdrawal (OR 1.38)	Urban & peri-urban areas more likely to use contraception (both modern & traditional)	Urban areas more likely to use withdrawal (OR 1.84)	Urban → more traditional use	Urban residence → more traditional use	Not specified	Not specified
**Reasons for non-use of modern contraception**	Husband wants more children, living in south/rural areas	Fear of side effects, lack of knowledge, modern methods considered inconvenient	Fear of side effects, limited access in rural areas, perception that modern methods are only for limiting children	Fear of side effects, husband’s opposition, lack of trust in modern methods	Husband opposition, fear of side effects, religious opposition	Not specified	Fear of side effects, particularly hormonal contraceptives	Lack of knowledge, fear of side effects, religious considerations, husband opposition
**Key policy implications**	Focus on young & high-parity women, expand access in southern region, involve husbands	Shift to social media, promote scientific NFP (Standard Days Method), involve men	Educate on side effects & management, improve counseling services, expand access in rural areas	Counseling sensitive to side-effect fears, target young & educated women	Involve husbands, discuss family planning during antenatal care & child welfare clinics	Target older women (>30 years), consider employment status	Promote Standard Days Method as natural, free, no side effects	Address knowledge gaps, fear of side effects, religious & husband opposition
